# Supplementary material for: Implementing high-throughput insect barcoding in microbiome studies: impact of non-destructive DNA extraction on microbiome reconstruction
Source: PeerJ. 2024 Sep 23;12:e18025. doi: 10.7717/peerj.18025 (PMC11426317; doi:10.7717/peerj.18025)
Supplement: Supplemental Information 9 — BioRender Publication Licensing which confirms the CC-BY permissions [file peerj-12-18025-s009.pdf]

BioRender Publication Licensing

The author **(Veronika Andriienko)**, has a Premium subscription to BioRender (biorender.com), and hereby BioRender grants **(PeerJ)** the permission to publish the figure(s), and only figure(s):

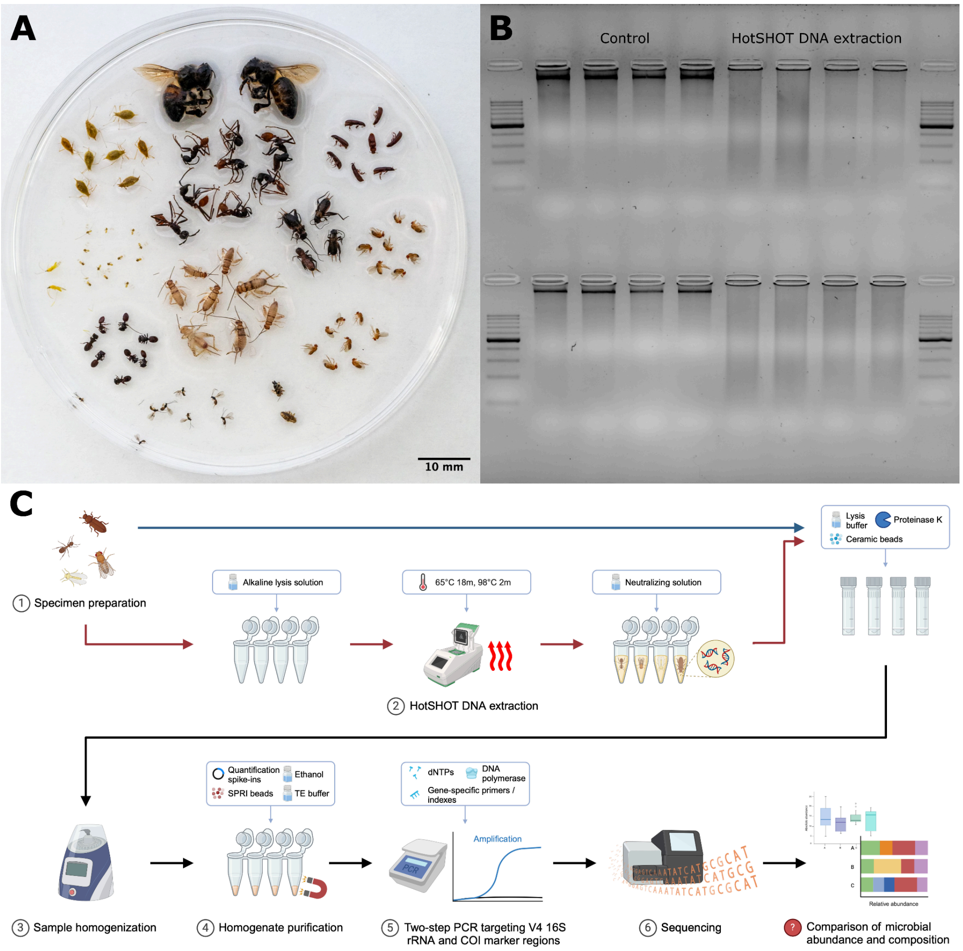

Figure 1. Insect species used in the study, DNA quality comparison, and study design. A. Representatives of the insect species used in the analysis. B. Gel electrophoresis comparison of DNA extracted from two insect species, Acyrthosiphon pisum (first row) and Aphis fabae (second row), between HotSHOT-treated and untreated samples. In the control samples, the DNA appears as long fragments, while in the HotSHOT-treated samples, the DNA appears as a smear of different fragment lengths typically shorter than 1kb, indicative of substantial degradation (ladder used: EURx Perfect™ 100-1,000 bp DNA ladder). C. Experimental workflow (Created with BioRender.com).

In the manuscript **(Implementing high-throughput insect barcoding in microbiome studies: impact of non-destructive DNA extraction on microbiome reconstruction)** on a CC-BY-NC-ND license. This includes the permission to publish the figure(s) titled above in all formats including print and digital versions. The manuscript must include an acknowledgement of "Created with BioRender.com" on all versions of the publication to acknowledge that the figure(s) have been created using BioRender.

This permission is exclusively granted for the figure(s) titled above and does not supersede or modify in any way BioRender's ownership, rights or license related to any of its intellectual property, as outlined in the BioRender terms and conditions located at https://biorender.com/terms/.

ADRIEL ABRERA, Customer Experience Associate

BioRender (Science Suite Inc.)

Adriel Abrera	July 29, 2024
Signature	Date
